# Self-Cross-Linking of Amine-Functionalized Polydimethylsiloxane on Metal Substrates with Humidity-Responsive Healing

**DOI:** 10.1021/acsapm.6c02244

**Published:** 2026-07-27

**Authors:** Kyujin Ko, Sarah Barber, Sazzadul A. Rahat, Benjamin M. Yavitt, Jonathan T. Pham

**Affiliations:** † Department of Chemical and Environmental Engineering, University of Cincinnati, Cincinnati, Ohio 45221, United States; ‡ Department of Mechanical and Materials Engineering, 2514 University of Cincinnati, Cincinnati, Ohio 45221, United States

**Keywords:** self-healing, PDMS, imine bonds, humidity responsive, dynamic networks

## Abstract

Polymer coatings require durability to maintain their protective and functional properties throughout their service life. This study presents amine-functionalized polydimethylsiloxane (PDMS) coatings that cross-link through metal substrate-catalyzed imine bond formation without additional curing agents. Amine-functionalized PDMS undergoes oxidative cross-linking on metal substrates, forming imine-linked networks with viscoelastic properties that are investigated as a function of curing time and temperature. The metal-catalyzed curing results in network formation across different metal substrates with varying cross-linking kinetics. The resulting imine cross-linked PDMS exhibits reversible viscoelastic transitions triggered by humidity, enabling the healing of surface damage. Upon exposure to elevated humidity, damaged coatings flow to close the gap, while subsequent thermal treatment restores the original modulus, demonstrating controllable healing recovery cycles. These findings establish a practical route for environmentally responsive polymer coatings with applications in protective barriers and flexible electronics, where damage repair is critical for continued functionality.

## Graphical abstract

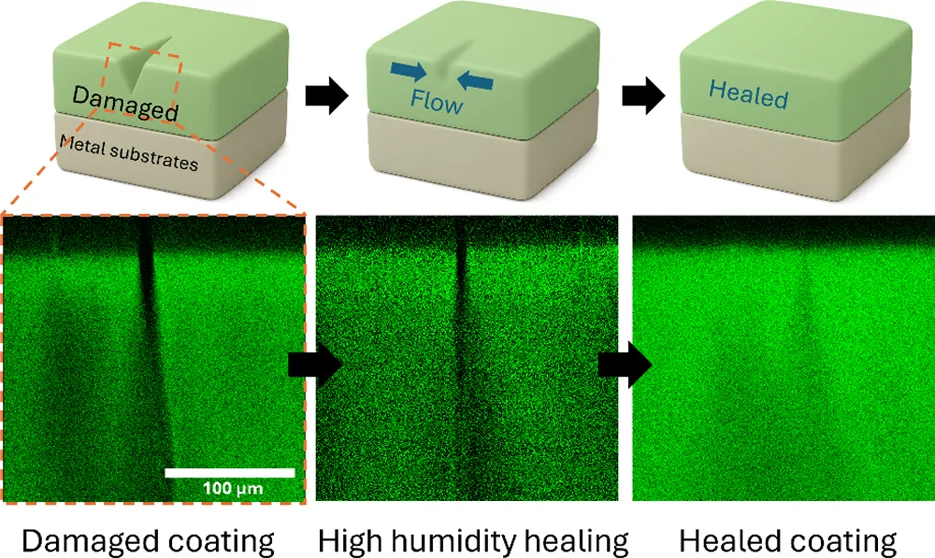

## Introduction

Polymer coatings are critical for a wide range of applications, from protective barriers to flexible electronics.^1–6^ In these cases, durability is essential because mechanical and chemical damage can lead to catastrophic failure of their function.^7–11^ For example, as a protective barrier, the coating should not have defects, scratches, or damaged regions that would allow external agents to reach the underlying substrate. One way to improve the lifespan of coatings is through the use of self-healing polymers.^12–15^ By employing materials that can heal, coatings can continue to be used after being scratched, as they will be able to repair that damage.^16–18^

Early self-healing strategies relied on extrinsic approaches, such as microencapsulated healing agents or vascular networks that release resins upon damage.^19,20^ However, these approaches are limited to one-time healing events and involve complex structural designs on larger length scales.^21,22^ More recent developments have instead focused on intrinsic self-healing, where reversible intermolecular bonds between polymer chains facilitate multiple cycles of damage repair.^23–25^ However, most self-healing studies require physically rejoining two cut pieces together to heal, which is then evaluated by tensile tests.^23,26–32^ Coatings are adhered to substrates and pose unique challenges, because once they are scratched, the separated surfaces are not easily rejoined. To address these limitations, healing can be induced through physical flow, chain diffusion, UV light, and shape memory effects.^33–38^ Among various triggers, humidity is an accessible and noninvasive route for coating repair. Previous studies demonstrate that moisture can trigger self-healing by inducing local plasticization or activating dynamic linkages, typically evaluated by rejoining completely severed bulk samples.^39–42^ While these reports establish the fundamental chemistry of moisture-driven network repair, the evaluations are primarily on bulk samples. Consequently, the healing behavior of a scratched coating under the constraints imposed by the substrate remains underexplored, presenting a barrier to its practical applications.

In addition to the durability and healability, the preparation of polymer coatings typically relies on mixing polymers and/or oligomers with cross-linkers;^43–46^ this can leave unreacted chemicals in the material that may leach out or compromise performance. Nonuniform network structures may also arise due to uneven cross-linker reactivity in high-viscosity systems. Hence, a route to create polymer coatings from reversibly cross-linked polymers, without additional cross-linker molecules, would be beneficial.

In this paper, we prepare imine-linked polydimethylsiloxane (imPDMS) coatings from amine-functionalized PDMS (amPDMS) via oxidation directly on metal substrates, without the need for additional curing agents. Imine bonds offer good mechanical recovery and responsiveness to humidity.^27,28^ By performing a healing process directly on the substrate, we consider the practical limitations associated with constrained coating repair. Through humidity-induced changes to the rheological properties, imPDMS coatings can heal scratches by transitioning from more solid to more liquidlike behavior, enabling gap closure and surface recovery (Figure 1). We investigate how the curing time on a metallic substrate controls the solidification and network formation of amPDMS, transforming it from a liquid to a viscoelastic solid. As the curing time increases, the modulus of imPDMS increases and the crossover frequency between the storage and loss moduli is extended. Our findings reveal that metal substrates can promote imine bond formation without external cross-linkers, while humidity serves as a control parameter for viscoelastic transitions inducing polymer flow for healing.

**Figure 1:**
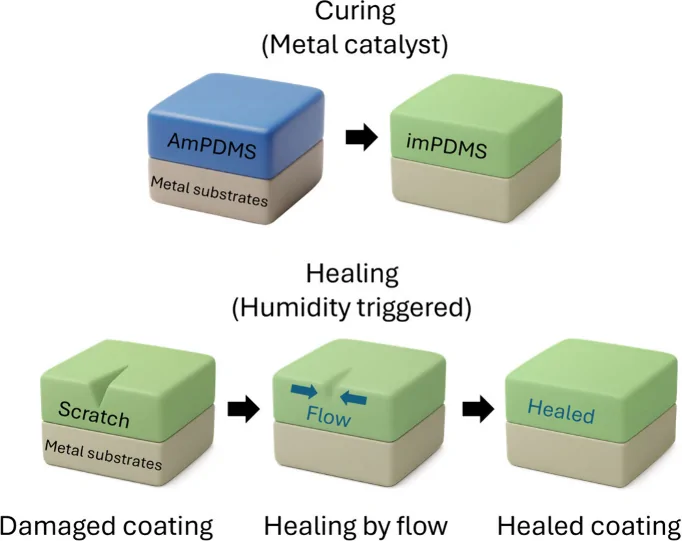
Schematic illustration of the curing process driven by the metal substrate catalyst and the healing process of a scratch triggered by humidity.

## Results and Discussion

### Thermal Curing

Amine-functionalized polydimethylsiloxane (amPDMS) undergoes time- and temperature-dependent cross-linking on copper substrates, without the need for conventional cross-linking agents (e.g., the addition of ketones or aldehydes). Oscillatory shear rheology shows a systematic transition from viscous liquid behavior to viscoelastic solid behavior in samples that are cured at constant temperature over the course of days. As an illustrative example, let us first consider the case of curing at 65 °C (Figure 2a). Curing for 1–3 days yields a viscous, fluid-like material. Upon increasing the curing time to 4 and 5 days, a plateau emerges across the frequency range, suggesting a transition to more observable solid-like behavior. The plateau modulus does not display a significant difference between 4 and 5 days. In addition to the modulus, a decrease in the crossover frequency (where storage and loss moduli cross) is observed with increasing curing times. Therefore, the material can still flow; however, the terminal relaxation process is pushed to longer time scales with increased curing. To test the effect of temperature, we measured the rheological properties after curing at 80 °C as well as 100 °C (Figure 2b and 2c). A similar trend is observed for these higher temperatures, where increasing curing time leads to a more solid material. The formation of a wide frequency range plateau in the modulus is observed with less curing days (shorter curing times) at higher temperatures, suggesting a rapid formation of the solid-like state. Additionally, the stable plateau modulus value appears to increase with curing temperature. For example, at 100 °C, the solidification process occurs within 1 day, having a higher plateau modulus compared to samples cured at lower temperatures for an equivalent time (Figure 2c). In general, lower curing temperatures and shorter curing times lead to viscous, fluid-like behavior, while higher curing times and temperatures promote more solid-like network formation.

**Figure 2:**
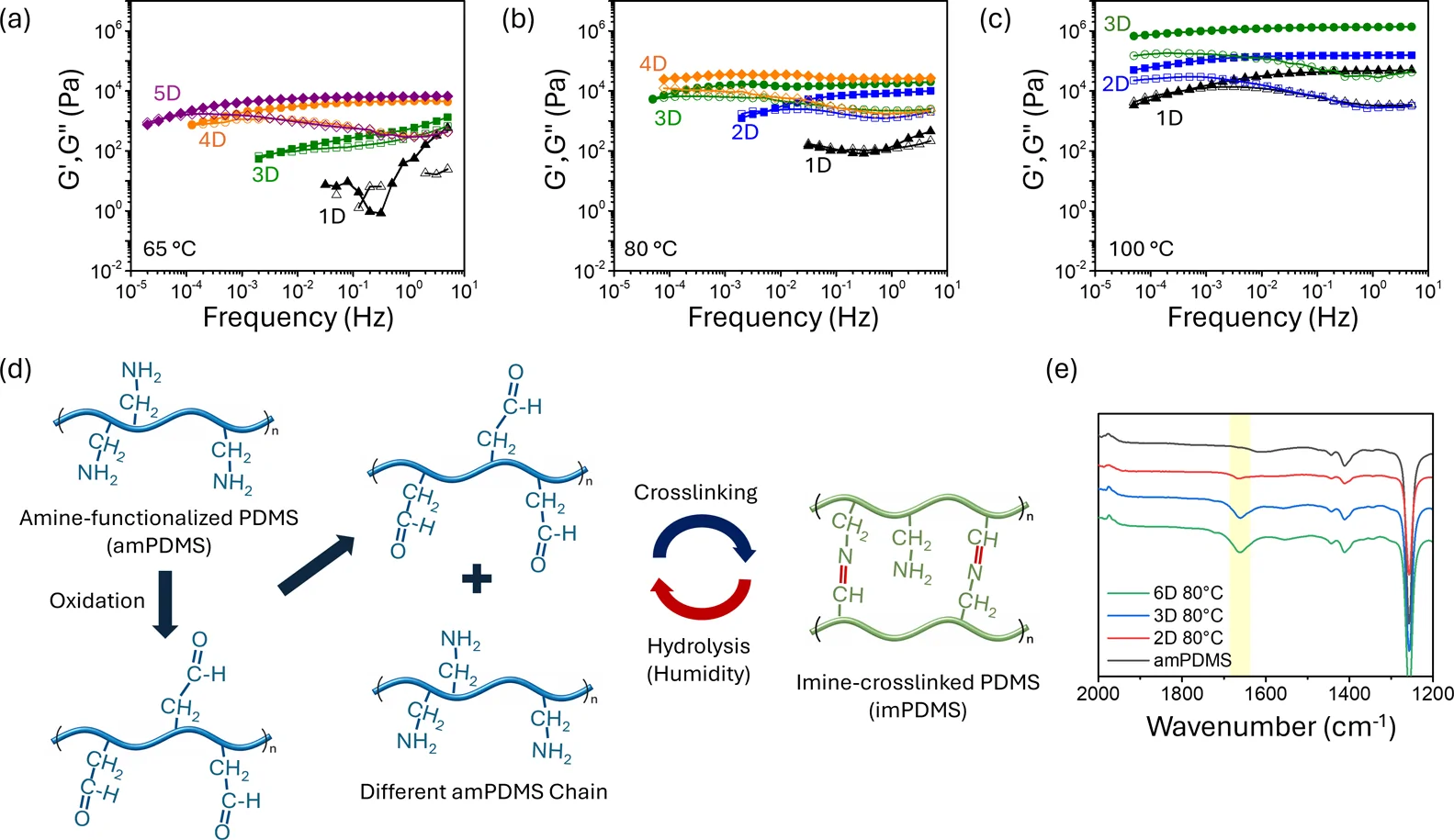
Curing of amPDMS on copper substrates: Linear rheology taken at room temperature (25 °C) of bulk polymers after curing at (a) 65 °C, (b) 80 °C, and (c) 100 °C for various curing times. G′ (closed data) and G″ (open data); (d) schematic of the reversible imine bond exchange mechanism; (e) FTIR spectra for different curing times after curing at 80 °C.

On the basis of these results, we hypothesize that this solidification is driven by a copper-catalyzed oxidative cross-linking mechanism, which leads to the formation of imine bonds from amines. According to the literature, this cross-linking might proceed through two potential pathways, both of which are facilitated by the metal catalyst to form imine bonds (details in Figure S1).^47–50^ The reversible nature of imine bond formation and hydrolysis can offer dynamic network behavior under appropriate environmental conditions^51–53^ (Figure 2d). In this system, the copper substrate functions as both a support and a heterogeneous catalyst, facilitating the oxidation of amine groups to form imine bonds.^49,54,55^ This aids in turning amine groups into imine linkages within the network, leveraging a mechanism demonstrated in the literature that uses copper powders or wires as heterogeneous catalysts.^56^

To confirm cross-linking, ATR-FTIR spectroscopy is performed on amPDMS first, which is then cured at 80 °C for varying curing times (Figure 2e). Major peaks corresponding to amine and imine functionalities, as well as those associated with the PDMS backbone, match well with what is found in the literature for similar materials.^57–59^ The absorption band near 1650 cm^–1^, associated with primary amines, gradually disappears with increased curing time. The absorption band emerges at 1661 cm^–1^ and intensifies over curing time, corresponding to the C=N stretch of the imine bond (N=CH). These spectroscopic observations show the formation of imine-cross-linked PDMS (imPDMS) networks during the curing process.^60,61^ Furthermore, to investigate cross-linking through the film thickness and the possibility of a cross-linking gradient, chemical and mechanical analyses of the imPDMS coating of the top surface and the bottom region are performed, as shown in Figure S2. We characterized the properties via FTIR and AFM indentation, which shows that the top surface has a lower imine peak intensity (via IR, Figure S2a), a 4× lower contact stiffness (via AFM, Figure S2c), and a correspondingly higher adhesion peak force (Figure S2d), when compared to the bottom region of the film. These results demonstrate that cross-linking is higher near the metallic surface compared to the top surface in contact with air, since it is in closer contact to the metal catalyst. Hence, a cross-linking gradient exists within the film. The FTIR data reported in this paper are from samples that combine top, middle, and bottom regions, serving as an average of properties.

### Humidity-Responsive Self-Healing Behavior

To evaluate the potential of using these materials as self-healing coatings, imPDMS cured at 80 °C is mechanically scratched to a depth of 200–300 μm using a razor blade and then placed in a humidity chamber for different durations. We use confocal microscopy to qualitatively visualize the healing process (closing of the scratch), using the natural fluorescence.^62,63^ We use imPDMS cured for 2 days as an example case. No healing is observed after 1 day in an environment of 40% relative humidity (RH) (Figure 3a), even up to 7 days (see Figure S3a). In contrast, exposure to 99% RH for the same duration results in scratch closure and recovery of the damaged region (Figure 3b). This humidity-responsive behavior demonstrates a transition of solid-like to liquid-like behavior, where moisture activates the self-repair process. To confirm the humidity-induced solid to flowable transition, we conducted a rheological analysis (Figure 3c). Exposing the sample to high humidity for 1 day results in a modulus reduction and increase in crossover frequency (light blue) compared to the as-cured material (blue). This transition into a weak plateau regime and increased crossover frequency results in the ability to repair a scratch by flow. On the other hand, the modulus slightly increases and the crossover frequency decreases over time at 40% RH (Figure S3c and d); this results in poor self-repair (Figure S3a,b).^33^

**Figure 3:**
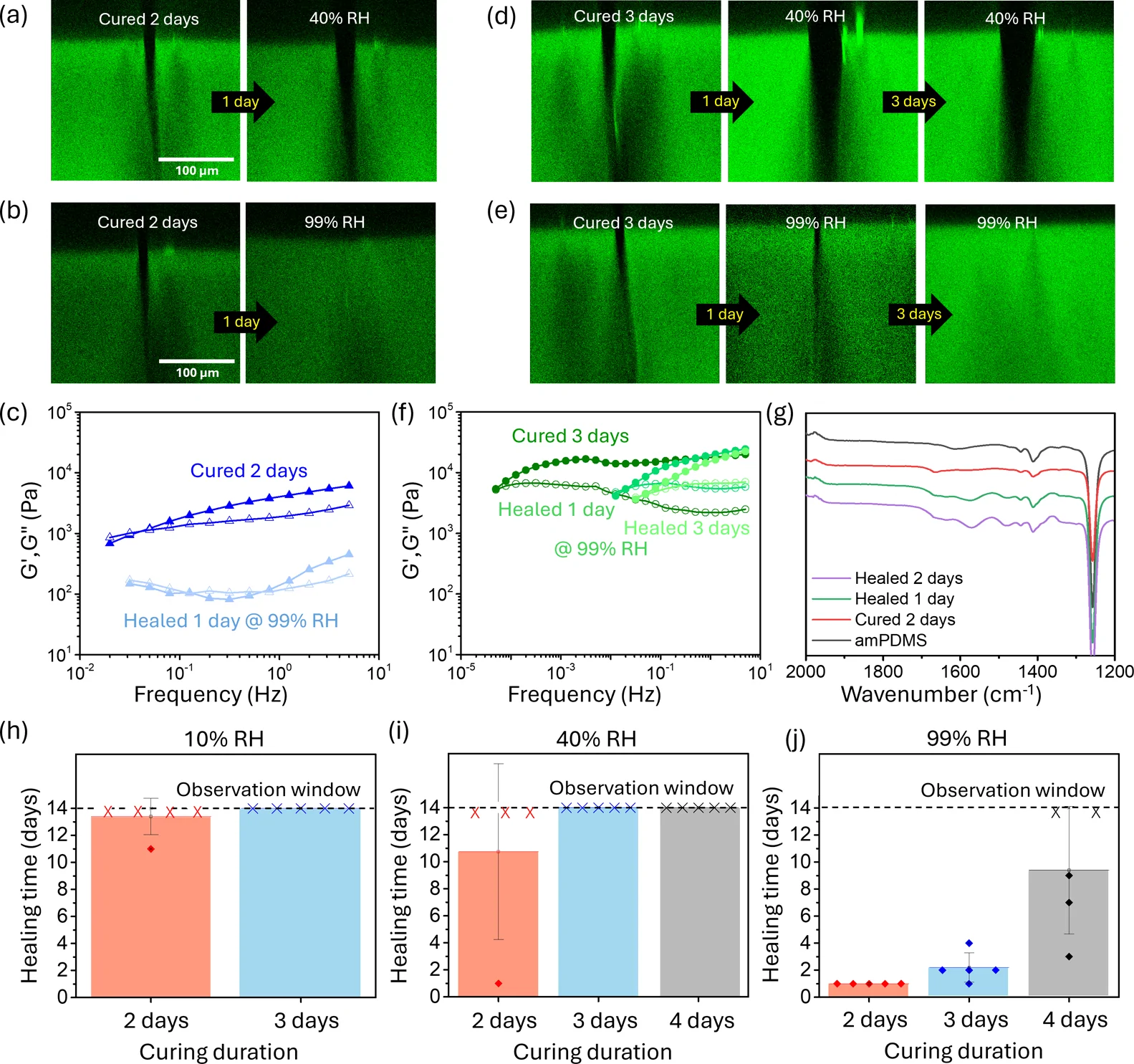
Humidity-triggered healing of scratched imPDMS coating. Cross-sectional confocal microscopy images of imPDMS cured at 80 °C for 2 days after being placed in (a) 40% RH and (b) 99% RH for 1 day. (c) Related rheological changes at 99% RH for the sample in (a) and (b). G′ (closed data) and G″ (open data). Confocal images for imPDMS cured at 80 °C for 3 days placed in (d) 40% RH and (e) 99% RH for 1 and 3 days. (f) Related rheological changes at 99% RH for samples in (d) and (e). (g) FTIR spectra of imPDMS cured for 2 days after being held at 99% RH. Overall healing time of imPDMS coatings at different curing duration and humidity levels of (h) 10% RH, (i) 40% RH, and (j) 99% RH for several samples to examine reproducibility. An X denotes that the sample did not heal after the 14-day observation window.

When samples are cured for longer durations, the modulus and relaxation time tend to increase (Figure 2), suggesting that it would take more time to repair. Increasing the curing time from 2 days to 3 days (i.e., higher cross-linking) shows a delayed healing process, requiring 3 days of healing time at 99% RH for complete scratch closure (Figures 3d,e and S3b for 40% RH), compared to 1 day for samples cured for 2 days. The longer healing time correlates with the lower crossover frequency (and longer relaxation time) of the more cross-linked network and is also likely associated with the higher initial modulus (Figures 3f and S3d for 40% RH). These results demonstrate that the healing rate is governed by a balance between cross-linking and moisture-induced flowability.

To confirm the hypothesized mechanism during healing, ATR-FTIR spectra are used to analyze imPDMS exposed to high humidity (Figure 3g). After high humidity exposure, the peak at 1661 cm^–1^ gradually disappears, signaling a loss of imines, while the peak at 1650 cm^–1^ intensifies, associated with a gain in amines. These results indicate that a reverse reaction occurs during the healing process, in which imines are hydrolyzed to amines, leading to un-cross-linking of the network. This reversible chemical transformation in the presence of moisture enables network rearrangement and more liquid-like behavior, affording macroscopic healing of a scratch.

To evaluate the reliability of the healing process, confocal microscopy images of imPDMS coatings are analyzed with healing defined as the complete closure of the crack and the recovery of a flat surface. Four to five independent replicates are tested for each curing duration and humidity level, and the observation period is maintained for up to 14 days (Figure S4). At a low humidity of 10% RH (Figure 3h), the majority of samples cured for 2 and 3 days do not exhibit healing within the 14-day observation window; only a single 2-day-cured sample shows recovery around day 11. A similar trend is observed at 40% RH (Figure 3i), where no healing occurred in the 3-day and 4-day cured samples. For the 2-day cured group, only one sample heals, while the remaining four samples show no observable change. These results indicate that low to intermediate humidity levels are insufficient to trigger the dynamic imine exchange for effective recovery, although there is some variability in our experiments. In contrast, the healing process accelerates at 99% RH for all of the curing durations (Figure 3j). All 2-day-cured samples achieve complete recovery within a single day. For the sample groups cured for 3 and 4 days, full recovery is reached in an average of 2 and 10 days, respectively. Collectively, these data demonstrate that high humidity acts as a trigger, facilitating reliable network rearrangement across all evaluated curing stages with some variability in the curing times.

### Potential Applicability for Coatings

We sought to further verify whether the coating successfully blocks external substances upon healing compared to the damaged surface. To address this question, an imPDMS coating is scratched, followed by putting a rhodamine B dye solution on top as a model external agent; this allows us to evaluate the effectiveness of healing for coating applications (Figure 4a). An imPDMS coating, cured for 2 days (Figure 4b), is scratched and exposed to 40% RH (Figure 4c) and 99% RH (Figure 4d) under the same conditions as those in Figure 3a–b. After 1 day, the high-humidity case appears to have closed the gap, while the low-humidity case does not. At 40% RH (Figure 4e), the scratch does not heal, allowing the rhodamine B dye (cyan) to penetrate into the crack. In contrast, confocal imaging clearly shows that the scratch successfully heals at 99% RH (Figure 4f), leaving the rhodamine B on the coating surface; this demonstrates both the protective capability and practical feasibility of the materials for self-healing coatings.

**Figure 4:**
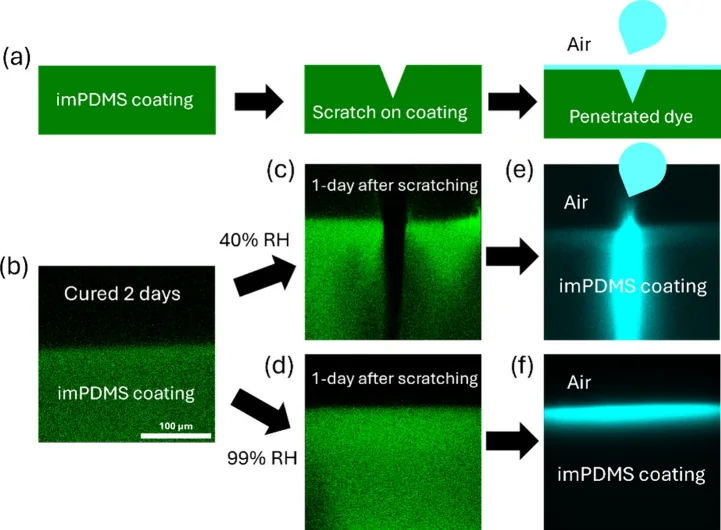
Confirming the protective function of healed coatings. (a) Schematic illustration of experimental procedure. A rhodamine B dye solution (cyan) is dropped onto the coating surface. (b) Cross-sectional confocal microscopy image of imPDMS coating cured for 2 days. The surface is then scratched and exposed to (c) 40% RH and (d) 99% RH for 1 day. The rhodamine B dye (e) clearly shows the crack at low humidity and (f) remains at the surface after healing at high humidity.

During the healing process in which the material becomes more liquid-like, the modulus also decreases; this can be an issue for coating applications where the original, as-made modulus is desired. From an applications perspective, it would be beneficial to have an easy process to resolidify the healed region quickly. To test this idea, we use a hand-held heat gun to heat the already-healed sample at 232 °C (450 °F) for 2 min (Figure 5a). This particular example is for a sample that was cured for 2 days at 80 °C. The complex modulus of the as-cured sample decreases with high-humidity healing, as expected, and then recovers the modulus similar to the original, as-made material (Figure 5b). This thermal modulus recovery demonstrates reversibility of the humidity-induced network breaking and enables cyclical healing and recovery operations. To test the potential for reusability, this humidity and heating cycle is repeated for three cycles (Figure 5b). This reversible trend remains consistent throughout the cycles, where humidity triggers a soft and healable state, while heat treatment restores the stiffness of imPDMS. The viscoelastic transition is further supported by tan δ values (Figure 5c). Upon humidification, tan δ increases, indicating a higher contribution of the loss modulus and a transition toward more liquid-like behavior. After heat treatment, tan δ returns to a lower value, confirming the recovery of more solid character. While a slight decrease in tan δ is observed in subsequent cycles, the coating consistently recovers its mechanical properties after each heat treatment. These results demonstrate that the imPDMS network maintains its ability to heal and solidify throughout multiple cycles. One could envision outdoor applications, where a natural humidity cycle could autonomously trigger healing during humid periods. For example, it is common in Cincinnati, Ohio, to have higher humidity at dawn and lower humidity in the afternoon (Figure S5), allowing self-regulated healing cycles without external intervention.

**Figure 5:**
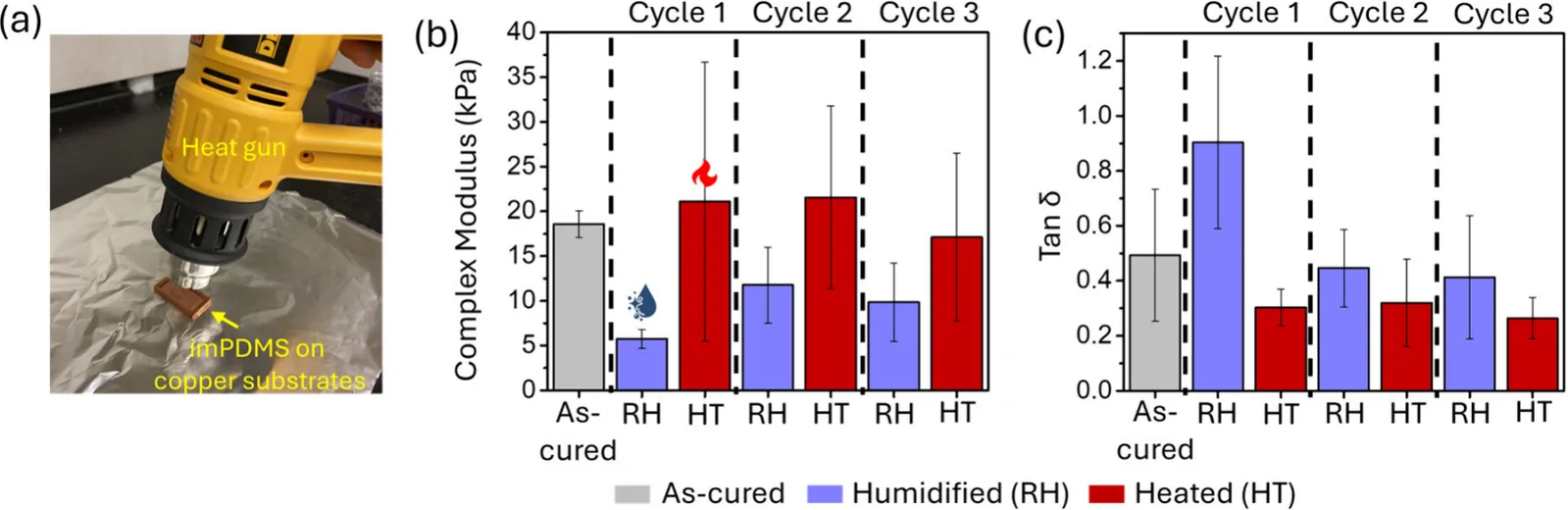
Modulus recovery. (a) Photograph of heat gun treatment to an already-healed imPDMS (cured 2 days at 80 °C). (b) Complex modulus and (c) tan δ of the imPDMS measured at 1 Hz over consecutive cycles of as-made, high-humidity, and heat-treated states.

## Conclusion

We prepared imine-linked PDMS (imPDMS) networks on metal substrates without additional cross-linking agents. A clear transition from liquid-like behavior to viscoelastic, solid-like behavior is observed with increasing curing time and temperature. We hypothesize that copper substrates function as a heterogeneous catalyst, facilitating amine oxidation to form imine linkages to create a polymer network. The imPDMS coating exhibits humidity-triggered healing of a scratch by activating flow of the material through a solid-to-liquid-like transition, enabled by imine hydrolysis. To quickly resolidify the material to the as-prepared state, a thermal treatment with a hand-held heat gun recovers the modulus, demonstrating controllable healing-recovery cycles. Furthermore, the healed coating is able to block a model external agent, confirming that the healing process restores not only the structural integrity but also the coating function. Further work is needed to quantify the relationship between metal substrate properties and cross-linking kinetics, as well as details on the limitations of curing and healing times. The current findings provide design principles for autonomous self-healing coatings driven by humidity.

## Methods

### Materials Preparation

Copper substrates (110 copper) from Grainger and McMaster-Carr with dimensions of 2 cm × 4 cm × 0.3 cm are machined to include a rectangular cavity, measuring 1.5 cm × 3 cm × 0.15 cm. The substrates are then cleaned with acetone and isopropanol (ACS grade, VWR) using an ultrasonic bath for 15 min. 20–25% aminopropylmethylsiloxane-dimethylsiloxane (Mw = 20000g/mol) was purchased from Gelest (AMS-1203) and used as is. 700 μL of the material is filled into the cavity of the metal substrates and then cured in an oven at temperatures of 65, 80, and 100 °C for varying durations of up to 5 days.

### Healing and Recovery

The surface of imPDMS on the metal substrate is scratched with a razor blade (scratch depth: 200–300 μm), and the samples are placed in a home-built humidity chamber at 10% relative humidity (10% RH), 40–50% relative humidity (ambient, denoted as 40% RH), and 99% relative humidity (99% RH). Relative humidity is continuously monitored in real time using a hygrometer (Veanic, measuring range: 10–99% RH with ±5%). No additional intervention is applied during the healing process. Cross-sectional images of scratched imPDMS are recorded over time using confocal microscopy. The humidity-exposed imPDMS is subjected to recovering the mechanical properties with thermal treatment at 450 °F (232 °C) for 2 min using an industrial heat gun (DEWALT, air volume: 17.7 CFM). The heat gun is positioned at a fixed distance of approximately 3 cm from the sample surface to ensure uniform heat distribution. During the thermal treatment, the samples were maintained in ambient atmospheric conditions. The ability for the coating to heal and remain a barrier to external agents is evaluated by applying an aqueous rhodamine B dye solution (50 μg/g, Merck KGaA) onto the surface and imaged with cross-sectional confocal microscopy. The overall experimental procedure for the preparation and healing evaluation of the imPDMS coating is schematically illustrated in Figure S6.

### Characterization

#### Chemistry

An attenuated total reflectance Fourier transform infrared spectrometer (ATR-FTIR, Thermo Nicolet 6700, SmartOrbit Diamond crystal, Thermo Scientific) is used in the wavelength range of 600 to 4000 cm^–1^ to investigate chemical bonds of amPDMS and imPDMS to investigate cross-linking and healing.

#### Rheology

A rheometer (TA Instruments Discovery HR-2) equipped with 8 mm aluminum parallel plates is used to study rheological behavior. Each sample is tested over a frequency range from 10 Hz to 10^–4^ Hz at 0.5% strain at room temperature. An amplitude sweep was conducted first to confirm this strain value was within the linear viscoelastic region.

#### Confocal Imaging

Cross-sectional images of imPDMS during healing are taken using an inverted confocal microscope (Leica SP8) equipped with a piezo-driven objective. A 20× air objective with a correction ring and a 488 nm laser source is used.^62,63^ Emission in the 500–600 nm range is collected using a high-sensitivity detector. The cross-sectional images are recorded at a resolution of 1204 × 1204 pixels.

#### Atomic Force Microscopy

A JPK Nanowizard 4a atomic force microscope is used to analyze the stiffness and adhesion force of imPDMS coatings. The spring constant and sensitivity of cantilevers were calibrated prior to attaching microspheres to the tipless cantilevers. The colloidal probe size in these experiments was ∼30 μm. The cantilever was approached toward the surface at a rate of 2 μm/s and indented to a target vertical deflection force of 500 nN, followed by retraction at the same rate.

## Supplementary Material



## Notes

Additional chemical reaction mechanisms, FTIR spectra, AFM data, confocal microscopy images, rheological data, and detailed experimental illustrations (PDF)
