# Supplementary material for: Self-Cross-Linking of Amine-Functionalized Polydimethylsiloxane on Metal Substrates with Humidity-Responsive Healing
Source: ACS Appl Polym Mater. 2026 Jul 27;8(15):13178–85. doi: 10.1021/acsapm.6c02244 (PMC13488139; doi:10.1021/acsapm.6c02244)
Supplement: Supplementary file 1 [file ap6c02244_si_001.pdf]

Supporting Information:

Self-crosslinking of amine-functionalized polydimethylsiloxane on metal substrates with humidity responsive healing

Kyujin Ko[1], Sarah Barber[1], Sazzadul A. Rahat[2], Benjamin M Yavitt[1,2], and Jonathan T Pham[1,2]*

[1]Department of Chemical and Environmental Engineering, University of Cincinnati, Cincinnati, OH 45221, USA

[2]Department of Mechanical and Materials Engineering, University of Cincinnati, Cincinnati, OH 45221, USA

Email: Jonathan.Pham@uc.edu

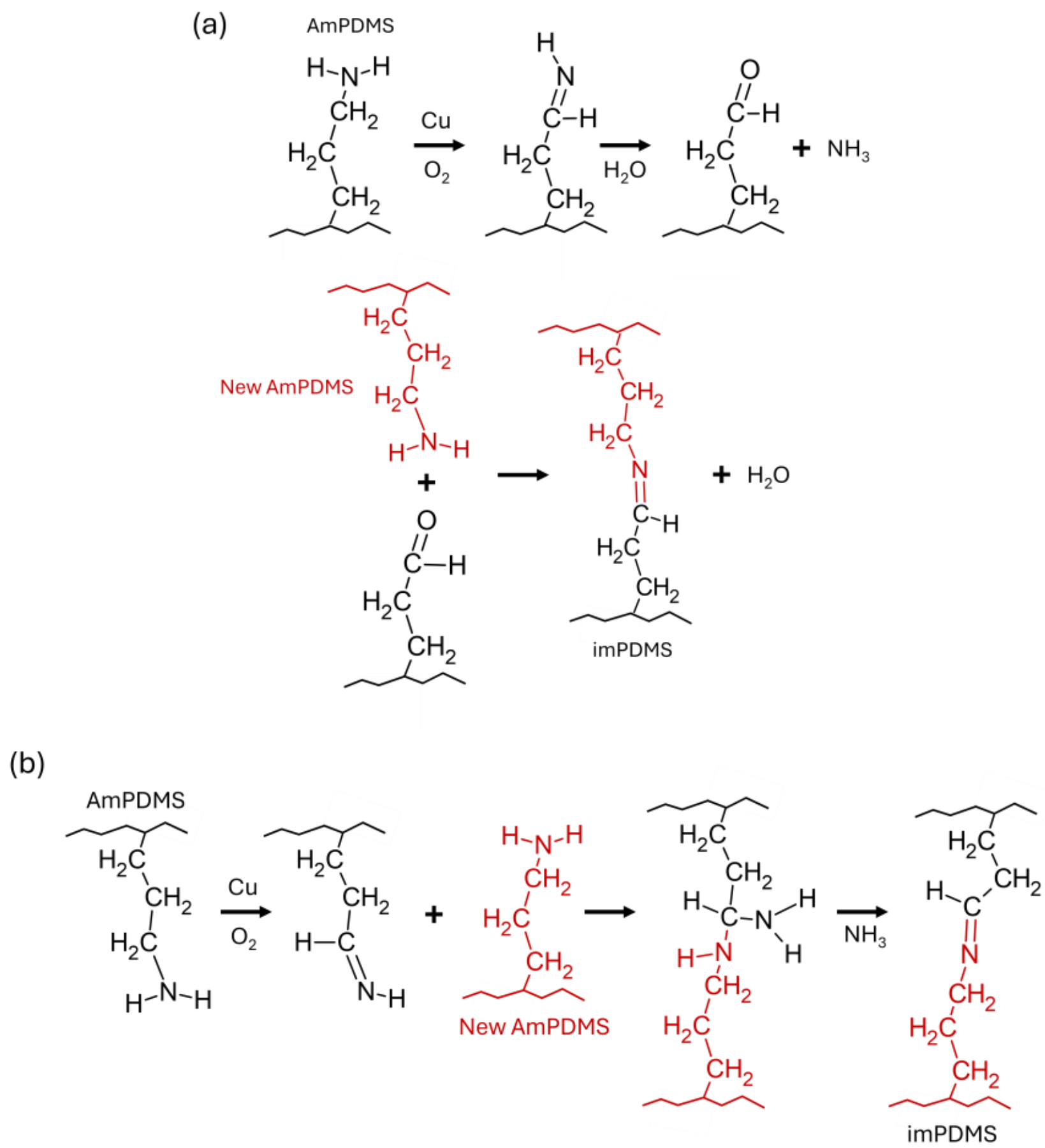

Figure S1. Possible reaction pathways for amine-to-imine crosslinking based on prior literature. Possible pathway 1 (a) is where the intermediate imine undergoes hydrolysis to form an aldehyde, which then reacts with another amine on a PDMS chain via condensation to form an imine bond (Fig. S1a). Possible pathway 2 (b) is that the amine group directly attacks the intermediate imine via a gem-diamine intermediate, followed by the elimination of ammonia (NH_3) to generate the final cross-linked imine network (Fig. S1b). [1-3]

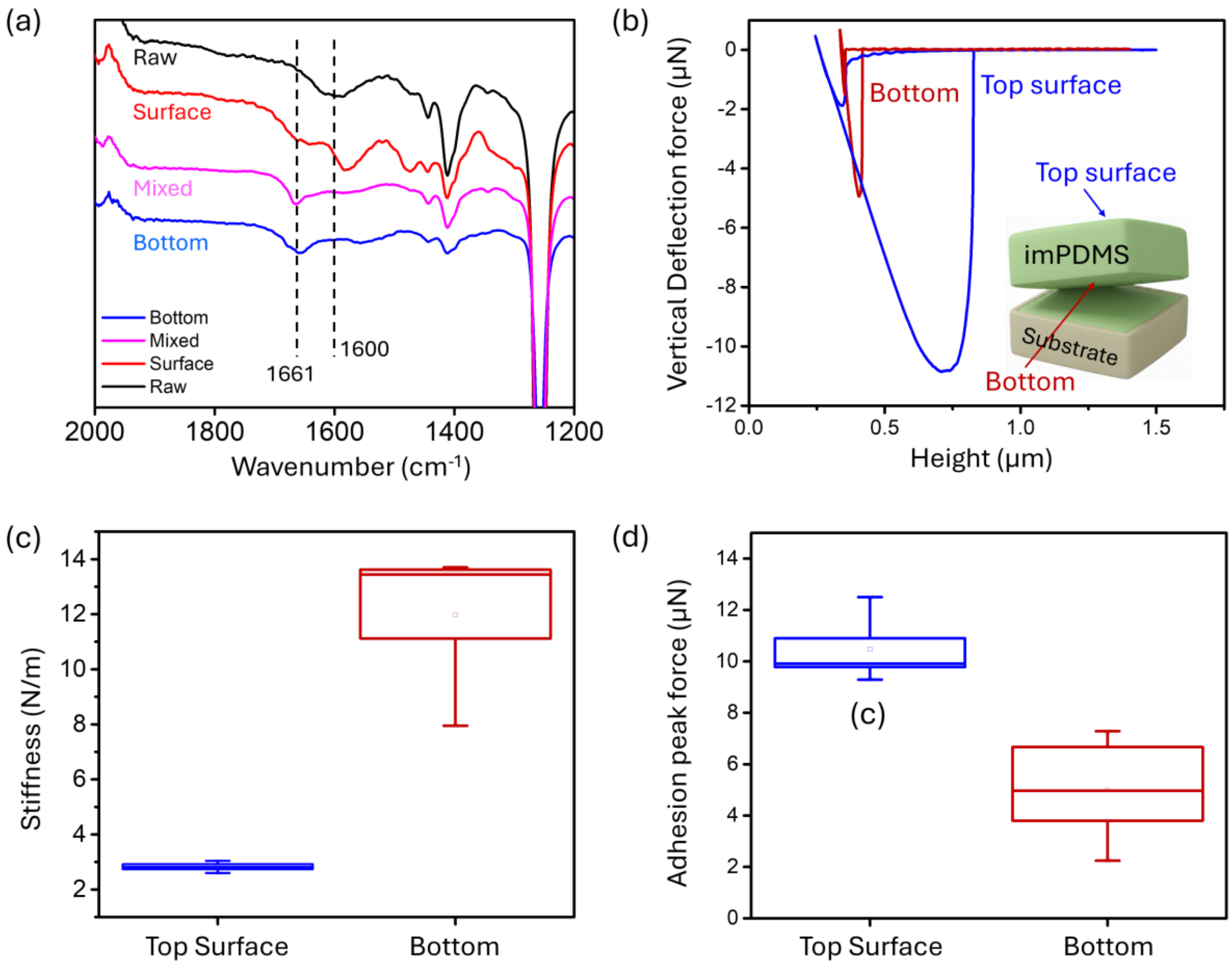

Figure S2. Chemical and mechanical characterization of the imPDMS coating top surface and bottom region. (a) FTIR spectra of the top surface, the bottom region, and a mixed sample of the top, middle, and bottom parts, labeled as Mixed. The top has a lower imine peak intensity than the bottom. (b) AFM force–distance curves obtained from the top (blue) and bottom (red) regions of the film. (c) Comparison of surface stiffness and (d) the peak adhesion force extracted from the AFM data. The slope of the approach curve in the contact region, spanning from the point of zero indentation to the maximum load, represents the contact stiffness. Higher adhesion is observed on the top compared to the bottom, which is consistent with the stiffness data; typically, softer materials display higher adhesive interactions through larger contact deformations.

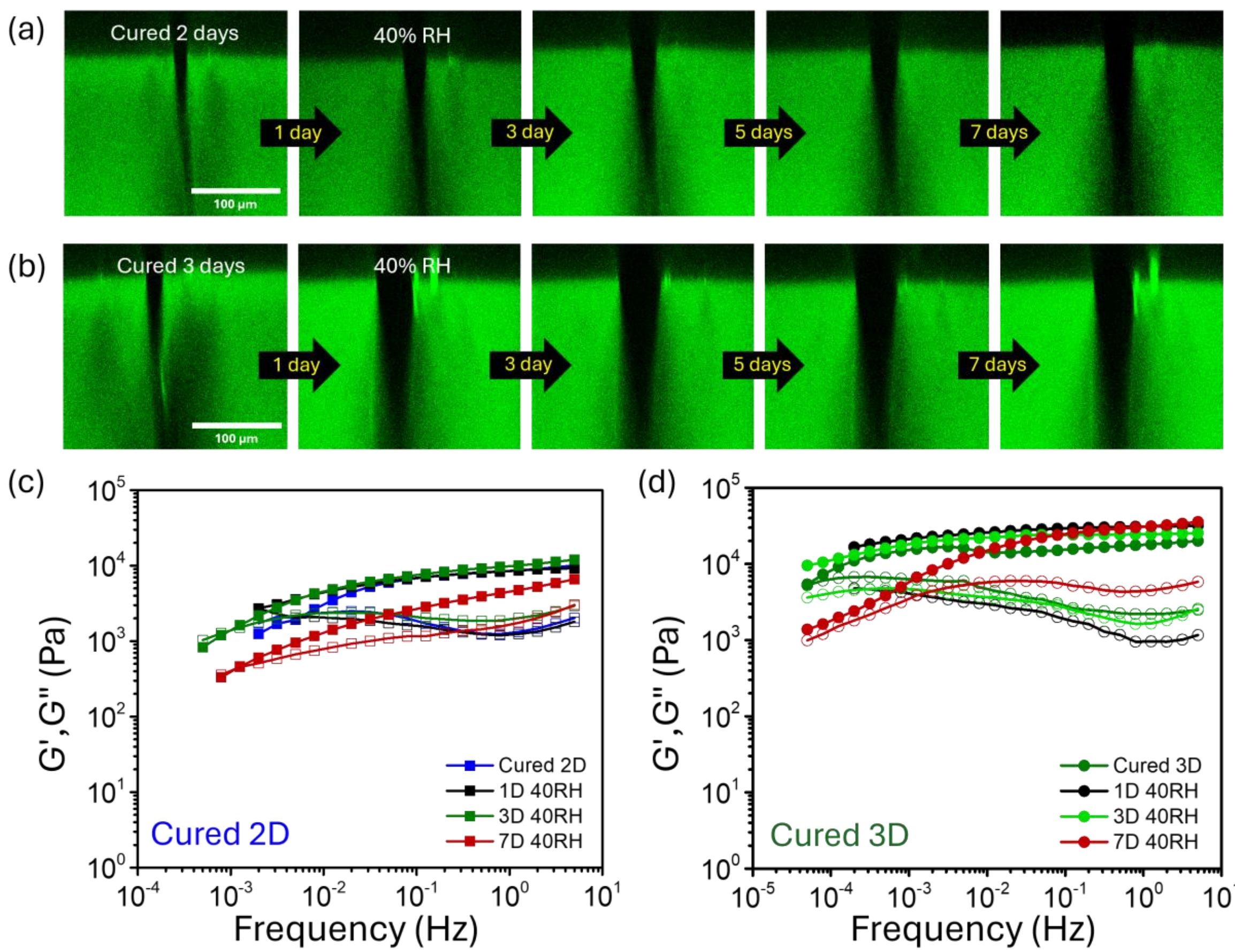

Figure S3. Confocal images of imPDMS cured for (a) 2 days and (b) 3 days at 40% RH; rheological changes at 40% RH for cured (c) 2 days and (d) 3 days imPDMS.

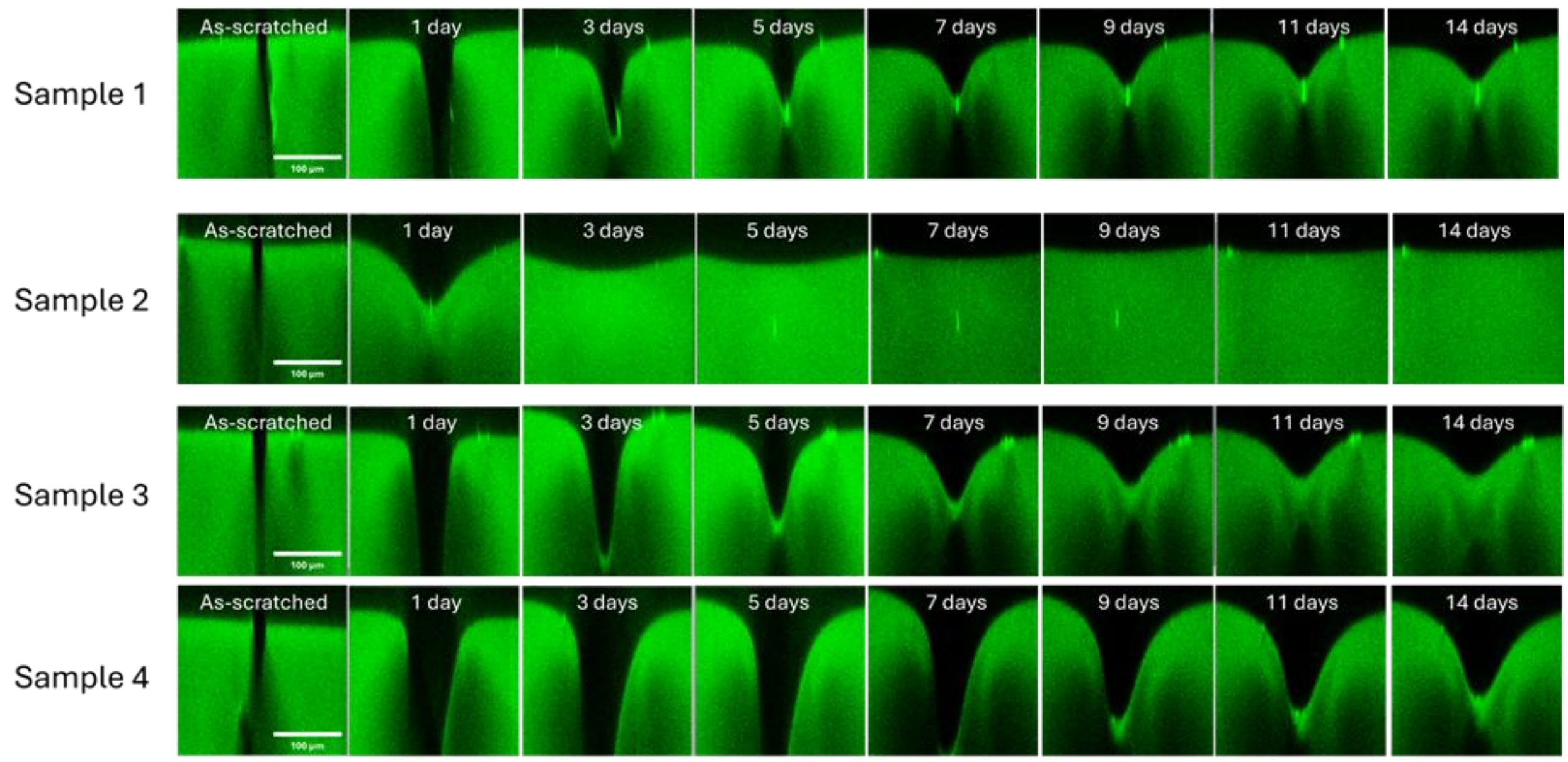

Figure S4. Confocal images showing the reproducibility of the self-healing process for imPDMS cured for 2 days. Four independent replicates (Samples 1–4) is observed at 40% RH over a 14-day period.

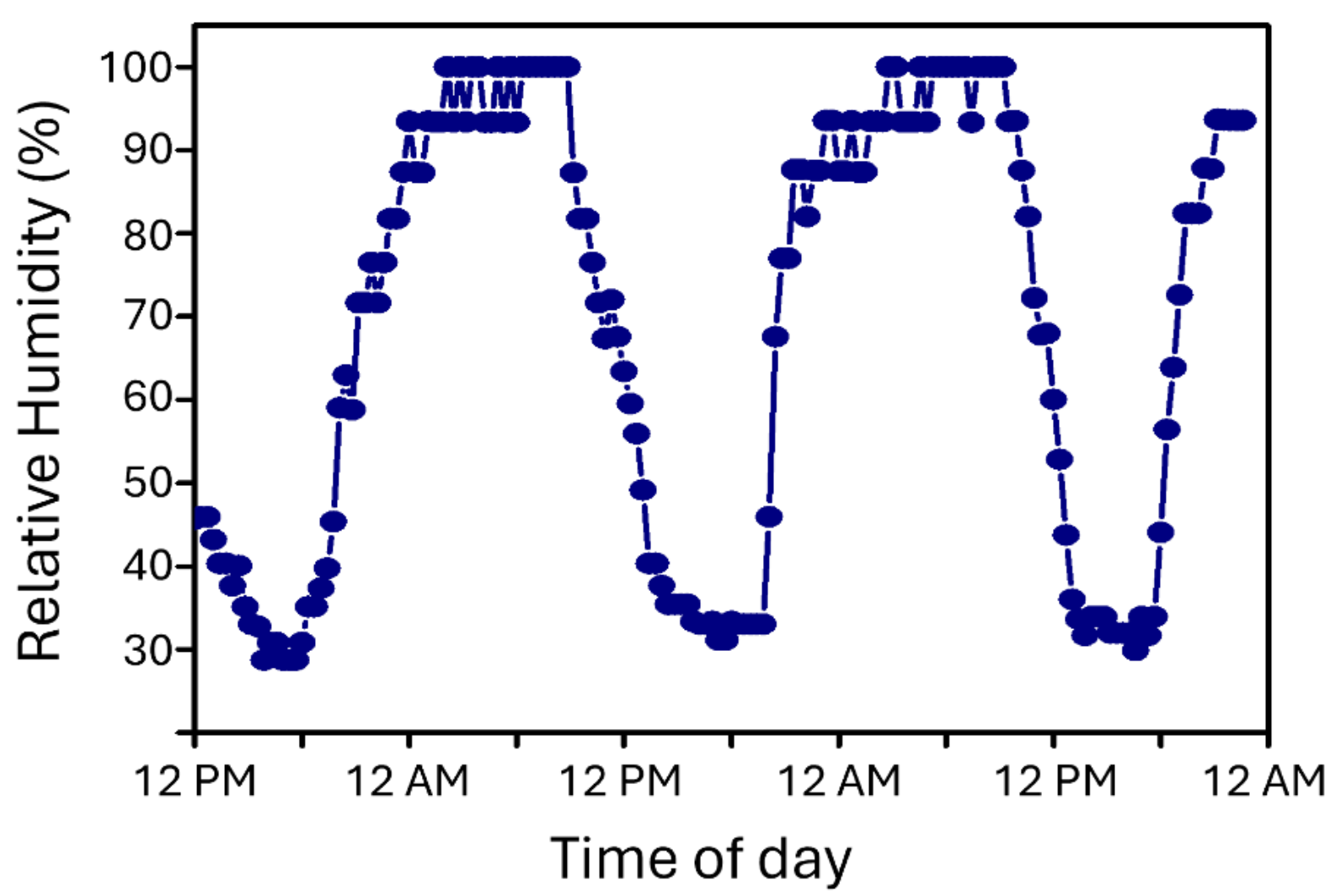

Figure S5. Relative humidity cycles over three days in September at Cincinnati West Airport. Data taken from National Weather Service.

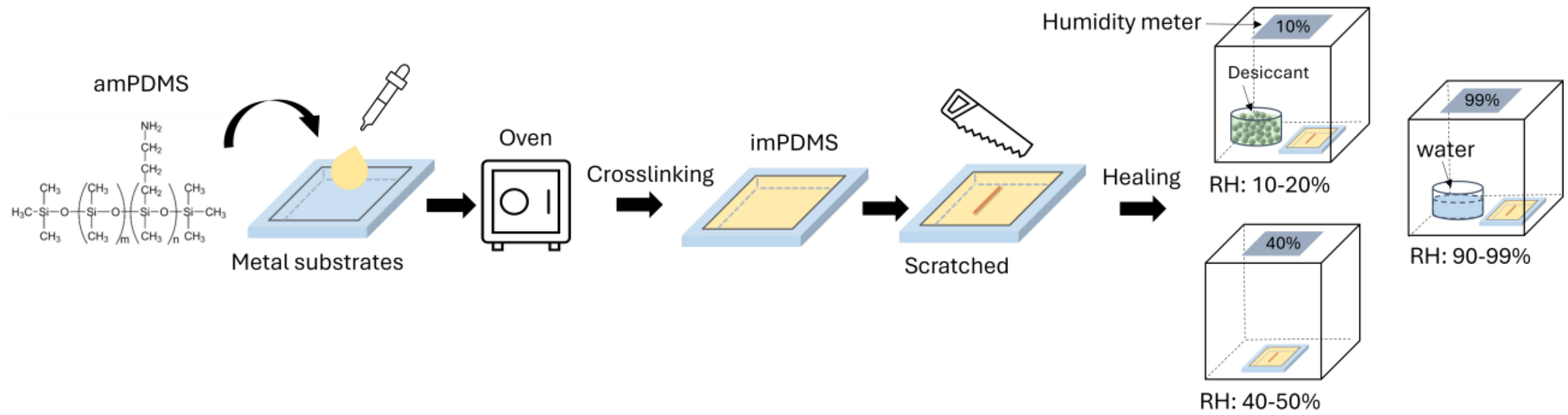

Figure S6. Schematic illustration of imPDMS curing from amPDMS and the self-healing test procedure under various humidity levels.

References

1. Largeron, M., Protocols for the catalytic oxidation of primary amines to imines. *European Journal of Organic Chemistry* **2013,** *2013* (24), 5225-5235.
2. Hoover, J. M.; Ryland, B. L.; Stahl, S. S., Mechanism of Copper(I)/TEMPO-Catalyzed Aerobic Alcohol Oxidation. *Journal of the American Chemical Society* **2013,** *135* (6), 2357-2367.
3. Markó, I. E.; Gautier, A.; Dumeunier, R.; Doda, K.; Philippart, F.; Brown, S. M.; Urch, C. J., Efficient, Copper-Catalyzed, Aerobic Oxidation of Primary Alcohols. *Angewandte Chemie International Edition* **2004,** *43* (12), 1588-1591.
